# Prevalence of anisometropia in children and adolescents

**DOI:** 10.12688/f1000research.73657.4

**Published:** 2022-06-08

**Authors:** Amélia F Nunes, Maria Batista, Pedro Monteiro

**Affiliations:** 1 Universidade da Beira Interior, Portugal, Covilhã, Portugal; 2 Health Sciences Research Centre (CICS-UBI), Universidade da Beira Interior, Covilhã, Portugal; 3 UBIMedical, Universidade da Beira Interior, Covilhã, Portugal; 4 Clinical and Experimental Center in Vision Sciences (CCECV), Universidade da Beira Interior, Covilhã, Portugal

**Keywords:** Pediatrics, Child, Teenager, Refraction, School vision

## Abstract

**Background:** This research was developed to study the epidemiology of anisometropia. It aims to estimate the prevalence of anisometropia in Portuguese children and adolescents at various educational stages, studying its association with sociodemographic variables.

**Methods: **Observational cross sectional study envolving 749 children and adolescents (from 3 to 16 years old) from the central region of Portugal. The refraction was performed with a paediatric, open field auto refractometer (PlusOptix), without cycloplegia and under binocular conditions, to determine the rate of anisometropia and its association with gender, study cycle and area of residence.

**Results: **The prevalence of anisometropia in the studied sample was 6.1%, varying from 2.9% in pre-school education to 9.4% in the 3rd study cycle. Myopic anisometropia was the most prevalent and hyperopic and astigmatic anisometropia showed identical proportions of occurrence. No statistical differences were found between genders or between areas of residence regarding the rate of anisometropia. Regarding spherical equivalent anisometropia, there was a pattern of variation that increased with the cycle of studies (p = 0.012), with myopic anisometropia being the main contributor to this variation.

**Conclusions:** This study found an increase in anisometropia with the educational stage. The high rate of anisometropia found in adolescents (9.4%) as well as the progressive increase in this rate throughout school progress (from 2.9% to 9.4%) suggests the need to extend the detection strategies of this condition beyond childhood.

## Introduction

Anisometropia is an ocular disorder characterized by an interocular difference (IOD) in refractive error. It represents a specific refractive condition where the two eyes of an individual, can have asymmetric eye growth. ^1^ This condition can occur in situations of myopic, hyperopic or astigmatic asymmetry and is strongly associated with the development of other eye changes such as aniseikonia, amblyopia, diplopia and strabismus. ^2^^,^^3^

Although there is no uniformly defined dioptric value for its clinical classification, an IOD in the spherical equivalent (SE) of 1 diopter or more is accepted as the threshold, for most authors. ^1^^,^^4^^–^^8^ However, even using this limit, the scientific literature presents a significant variation in the prevalence values of anisometropia in terms of age, gender and ethnicity. ^4^^,^^5^^,^^8^^,^^9^ Factors associated with lifestyle and educational level have also been referred to as risk factors for anisometropia. ^7^^,^^8^^,^^10^

Early detection and early treatment is crucial to prevent permanent visual loss. Although it is not clear what is the ideal age to perform the correction, in order to guarantee an ideal visual development and maturation, the early correction of anisometropia is important. ^11^ This prevents the development of other changes such as aniseikonia, amblyopia and strabismus, ^3^^,^^12^^,^^13^ and even in small degrees (<1D) facilitates emmetropization. ^11^ It also improves quality of life, reducing or eliminating symptoms of visual discomfort. In this way, visual screening at a young age is useful in identifying who is most likely to benefit from early optical correction or preventive treatment. ^11^^,^^14^^,^^15^

The clinical methods used to characterize anisometropia are refractive techniques, with autorefraction, using Plusoptix, one of the most recommended techniques for screening activities. ^16^^,^^17^ This instrument allows quantifying the refractive error in which it is possible to obtain a very similar value to that obtained by cycloplegic refraction ^17^^–^^19^ and with excellent precision in anisometropia signalling. ^19^

Studies on the prevalence of anisometropia focus on children or adults, with less research being found in adolescence.

The aim is to estimate the prevalence of anisometropia (spherical and astigmatic) and to analyse its pattern of variation in a sample of children and adolescents, from preschool education (from three to six years old) to the various cycles of basic education (from the 1 ^st^ to the 9 ^th^ school year in Portugal, from six to fifteen years old).

## Methods

This is an observational cross sectional study to evaluate the prevalence of anisometropia in children and adolescents. Data from the Portuguese Census 2011, showed a population of 319284 from 0 to 14 years old in the central region. ^20^ For a confidence level of 95% and a 5% margin of error, taking into account that the prevalence of the studied condition is unknown, it was fixed at 50% to obtain a large enough sample size. The result was a minimum of 384 subjects.

The data collection took place in five schools of the central region of Portugal, including all students that were authorized to participate by their legal guardians, between October 2018 and February 2019. Participants were 749 children and adolescents aged between three and sixteen years. Students data for which it was not possible to obtain refraction were excluded, due to technical issues associated with the performance of the instrument (presence of strabismus, opacities, retinal anomalies or when the refractive error exceeded the instrument's measurement limit - spherical measurement range or cylindrical from −7.00 to +5.00D) or due to lack of cooperation from the participant. Only two participants were excluded.

The refractive error was measured with the paediatric auto refractometer model A09 by PlusOptix, Nuremberg, Germany, by the average of three consecutive measurements, binocular and without the use of a cycloplegic. The PlusOptix allows measuring the refractive error in open field simultaneously in both eyes and under the same conditions, in a fast, easy, safe, non-invasive way, at a distance of one meter from the subject's eyes.

### Sample characterization

In Portugal the compulsory education includes basic education, which is divided into 3 cycles, followed by the secondary education. The 1 ^st^ cycle of basic education includes the 1 ^st^ to 4 ^th^ school year (ages 6 to 10), the 2 ^nd^ cycle includes the 5 ^th^ and 6 ^th^ year (ages 10 to 12) and the 3 ^rd^ cycle includes the 7 ^th^ to the 9 ^th^ year (ages 12 to 15). The sample under study had students from preschool to the 3 ^rd^ cycle of basic education and was characterized according to gender, area of residence and cycle of studies. Rural and urban areas classification was based on the information provided by the municipalities, considering the residence area of each participant. Regarding the use of glasses, 177 participants wore glasses or contact lenses and 572 did not use any type of optical correction. Table 1 summarizes the characteristics of the sample.

**Table 1.** Sample characteristics.

Factor		Sample dimension n (%)	Wearing glasses		
			Yes (n)	No (n)	missing
Gender	Male	399 (53.3%)	80	318	1
	Female	350 (46.7%)	95	254	1
Residence area	Rural	320 (42.7%)	69	249	2
	Urban	423 (56.5%)	104	319	0
	Missing	6 (0.8%)	-	-	-
Study stage	Preschool	103 (13.8%)	3	100	0
	1 ^st^ cycle	231 (30.8%)	36	195	0
	2 ^nd^ cycle	181 (24.2%)	44	136	1
	3 ^rd^ cycle	234 (31.2%)	92	141	1

### Data analysis

In order to calculate the average of the three refractive measurements, the power was converted from its spherical-cylindrical form to its power vector representation, described by Thibos, ^21^ using the following expressions:

$$\mathrm{SE}=S+\frac{C}{2}$$ (1)

$$J_{0}=\left(-\frac{C}{2}\right)\cos\left(2\alpha \right)$$ (2)

$$J_{45}=\left(-\frac{C}{2}\right)\sin\left(2\alpha \right)$$ (3)

Where SE represents the spherical equivalent; *J*_0_ represents Jackson's crossed cylinders on the 90° or 180° axis, which stands for the amount of direct or indirect astigmatism; *J*_45_ represents Jackson's crossed cylinders on the 45° or 135° axis, which stands for the amount of oblique astigmatism; *S*, *C* and *α* represent the spherical, cylindrical (in negative form) and cylinder axis component, respectively, of the auto refractometer measurement.

### Refractive state classification

The participants were classified in emmetropes, myopes, hyperopes, astigmats or anisometropes, according to the average value of the auto refractometer. In order to carry out this classification, the criteria recommended for the auto refractometer used were applied ( Table 2).

**Table 2.** Classification criteria for refractive state. ^16^

Refractive state	< 6 years old	≥6 years old
Emmetrope	−1.00D < SE < +1.25D	−1.00D < SE < +1.00D
Myope	SE ≤ −1.00D	
Hyperope	SE ≥ +1.25D	SE ≥ +1.00D
Astigmat	|C|≥1.00D	|C|≥1.25D
Anisometrope	|IOD|≥1.25 (SE or C)	

### Anisometropia classification

The absolute value of IOD of the refractive error in terms of SE was designated as spherical anisometropia (SA), the absolute IOD in astigmatism was designated as meridional anisometropia (MA),

$$\mathrm{SA}=\left|{\mathrm{SE}}_{\mathrm{RE}},-,{\mathrm{SE}}_{\mathrm{LE}}\right|$$ (4)

$$\mathrm{MA}=\left|C_{\mathrm{RE}},-,C_{\mathrm{LE}}\right|$$ (5)

where RE refers to the right eye and LE to the left eye.

When there was no IOD in the spherical equivalent and the absolute IOD was only in the astigmatic component, it was designated by simple meridional anisometropia (sMA). The anisometropia classification was done according to the cutoff points referred to in Table 2. The presence of at least one of the previous conditions was designated as total anisometropia (TA). Low anisometropia was considered for IOD values below 2.00D, high anisometropia for values between 2.00D and 6.00D and very high anisometropia for IOD values above 6.00D.

According to the type of refractive error, anisometropia was classified as myopic, when both eyes were myopic or when one eye was myopic and the other was emmetropic; hyperopic, when both eyes were hyperopic or when one eye was hyperopic and the other emmetropic; antimetropic, when one eye was myopic and the other hyperopic; simple meridional anisometropia when there was no SA, but there was MA.

### Statistical analysis

A descriptive statistical analysis was carried out, using SPSS version 26 package, characterizing the sample in the variables of interest, sociodemographic and refractive, presenting means and standard deviations, frequencies and percentages both in the whole of the sample and also according to several stratifications to which it was subjected.

In all the sociodemographic factors in which the sample was categorized, groups with a large size (n > 30) were obtained. The proportion of subjects with anisometropia was analysed according to gender, area of residence and school cycle, and through the Chi-square test, it was evaluated whether these variables were associated with the occurrence of anisometropia in the studied population.

All the results of the statistical inference tests were interpreted to a 95% confidence level, that is, the significance level of 0.05 was used.

### Ethics approval

This study was conducted in accordance with the principles of the Declaration of HELSINKI and written informed consent as obtained from parents of each participant in the study. It was approved by the Ethics Committee from Universidade da Beira Interior (CE-UBI-Pj-2019-043).

### Consent to participate

Written informed consent was obtained from parents or legal guardians of all participants.

## Results

### Refractive state

**Figure 1.**
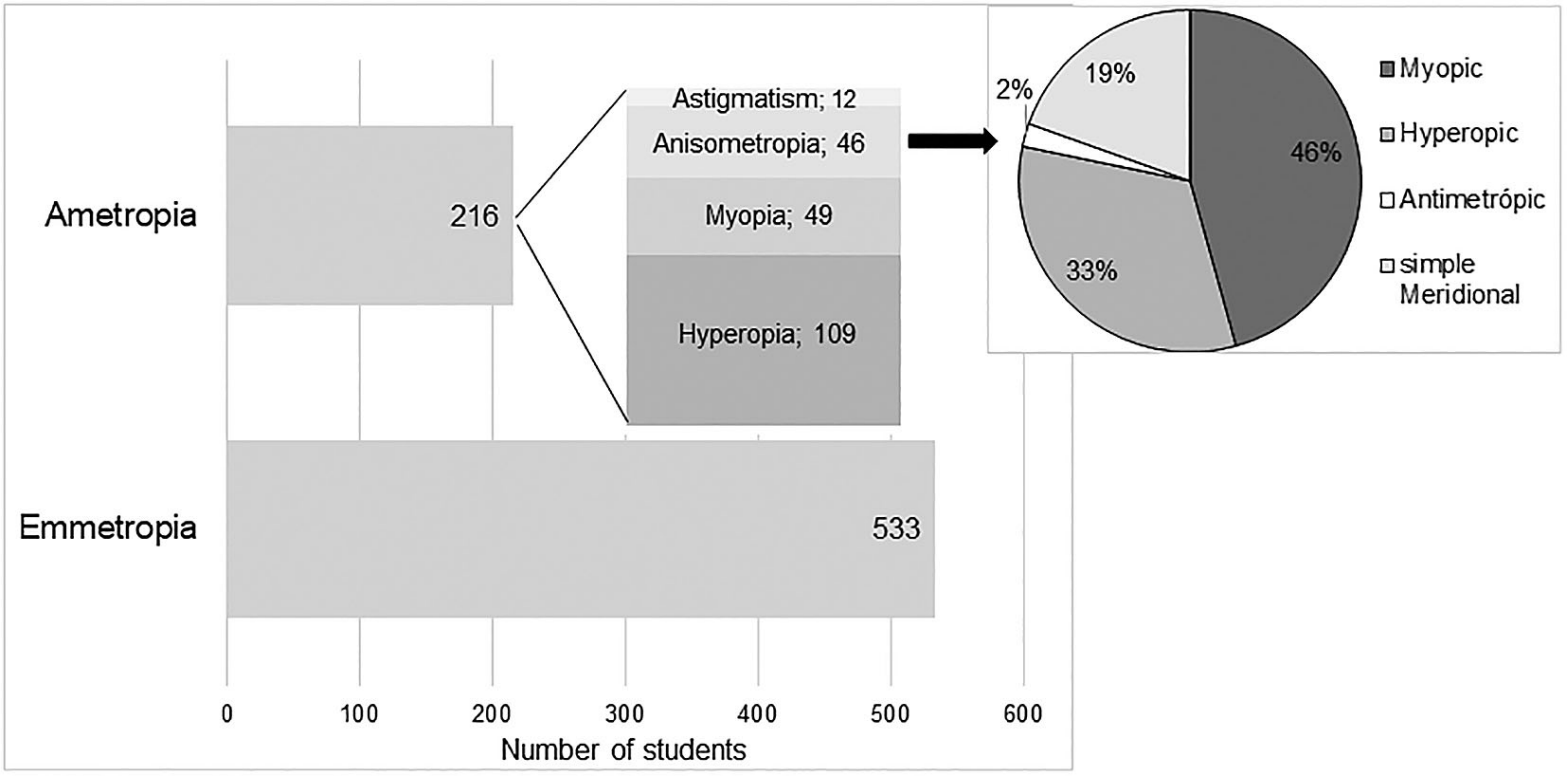
Refractive error distribution.

According to the classification criteria previously defined for the classification of refractive state, it was concluded that in the study sample 71.16% (n = 533) were emmetropic. Among subjects with significant refractive error (n = 216), it was found that hyperopia was the most prevalent refractive error (n = 109, corresponding to 14.6% in the studied population) followed by myopia (n = 49, corresponding to 6.5% in the population), anisometropia (n = 46, corresponding to 6.1% in the population) and astigmatism (n = 25 where 12 were cases of simple astigmatism and 13 compound astigmatism). Figure 1 shows graphically the distribution of the different refractive states in the study sample. The representation of astigmatism, refers only to the occurrence of simple astigmatism, without a significant SE. The mean values of refractive errors, according to the previous classification, are shown in Table 3.

**Table 3.** Mean values and standard deviation of refractive errors of subjects with significant ametropia (right eye data).

Refractive group	Wearing glasses (n)		Spherical equivalent (D)		Cylindrical component (D)	
	Yes	No	Mean ± SD	Range [min; max]	Mean ± SD	Range [min; max]
Myopia	44	5	−2.67 ± 1.32	−5.79; −0.92	−0.39 ± 0.33	−1.29; 0.00
Hyperopia ^§^	22	85	+1.46 ± 0.59	+1.00; +5.25	−0.41 ± 0.47	−2.50; 0.00
Simple astigmatism	6	6	+0.29 ± 0.55	−0.83; +1.21	−1.50 ± 0.33	−2.07; −1.14
Anisometropia ^ϯ^	37	8	Interocular difference			
			1.53 ± 0.82	0.00; 3.79	0.78 ± 0.85	0.00; 3.9

^§^ Two cases without data regarding the use of glasses.

^ϯ^ One case without data regarding the use of glasses.

### Anisometropia

Anisometropia was identified in 46 participants (6.1% of the studied population). Regarding the use of glasses, it was observed that eight cases did not use any type of optical correction.

According to the magnitude of the refractive error, no child was found with very high anisometropia, 15 were registered with high anisometropia and 31 with low anisometropia, that is, of the anisometrope subjects, most (67.4%) had low anisometropia. The dispersion of interocular diference in SA and MA is shown in Table 3.

In the classification of anisometropia according to the type of refractive error, 37 subjects (corresponding to 4.9% in the studied population) were found with SA, integrating 21 with myopia, 15 with hyperopia and 1 with antimetropia; 15 subjects (corresponding to 2% in the population) with MA, and only 9 of these did not have SA, and were classified as simple meridional anisometropia (sMA). This distribution is represented graphically in Figure 1. It is possible to observe that myopic anisometropia was the most prevalent (46%), followed by hyperopic anisometropia (33%) and sMA (19%). Antimetropic anisometropia was the least prevalent (2%).

### Influence of sociodemographic variables

The study of the influence of sociodemographic variables in the occurrence of anisometropia is presented in Table 4. The Chi-square test indicates that there was no association between MA and any of the factors under analysis.

**Table 4.** Relationship of gender, area of residence and study cycle, in anisometropia.

	Gender				Chi-square test
	Female (350)		Male (399)		
	N	%	N	%	*P*
TA	25	**7.1**	21	5.3	0.29
SA	21	**6**	16	4	0.21
MA	8	**2.3**	7	1.8	0.60

	Area of residence				Chi-square test
	Rural (320)		Urban (423)		
	N	%	N	%	*p*
TA	22	**6.8**	23	5.4	0.42
MA	7	**2.2**	8	1.9	0.78
SA	18	**5.6**	18	4.3	0.39

	School stage								Chi-square test
	Preschool (103)		1 ^st^ cycle (231)		2 ^nd^ cycle (181)		3 ^rd^ cycle (234)		
	N	%	N	%	N	%	N	%	*p*
TA	3	2.9	10	4.3	11	6.1	22	**9.4**	0.06
MA	2	1.9	3	1.3	6	**3.3**	4	1.7	0.52
SA	1	1.0	9	3.9	7	3.9	20	**8.6**	0.012 *
*p*_(adjust)_	0.36		<0.001 *		<0.001 *		0.015 *		

TA – Total anisometropes (spherical and meridional); SA - spherical equivalent anisometropia; MA – meridional anisometropia. The highest rates are shown in bold. Multiple comparisons with Chi-squared test for study cycles (Z: adjusted standardized residual to Chi-Square and *p* adjusted to Bonferroni correction).

* Significant at the 0.05 level.

No significantly different occurrence of anisometropia was found according to gender or area of residence (p > 0.05). In relation to the school, there was a pattern of variation that increased with the cycle of studies, ranging from 2.9% in preschool education to 9.4% in the 3 ^rd^ cycle of studies, however this association was only statistically significant for SA (p = 0.012), where there was a rate of 1% in preschool education and 8.6% in the 3 ^rd^ cycle. It should be noted that the study cycle is dependent on age.

**Figure 2.**
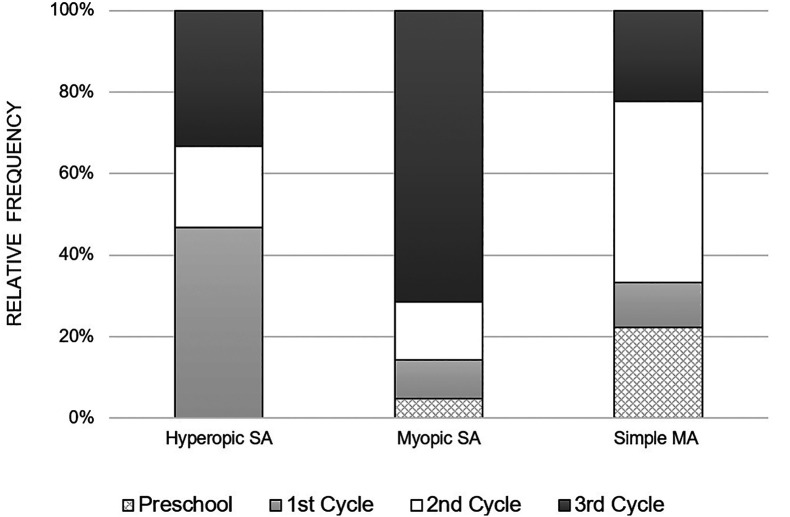
Relative frequency of the variation of the various types of anisometropia, with the school cycle.

Figure 2 illustrates the distribution of anisometropia according to the type of refractive error, for each school cycle. It is observed that myopic anisometropia was present in all study cycles, registering a considerable increase from the beginning of school, that is, from the 1 ^st^ cycle to the 3 ^rd^ cycle. On the other hand, hyperopic anisometropia was manifested on a larger scale in children of the 1 ^st^ cycle, with a lower occurrence in the following cycles. As for the simple MA, it was present in all study cycles and there was no specific pattern in its variation with the study cycle progress.

## Discussion

An anisometropia rate of 6.1% was found, considering the spherical and meridional anisometropia. It was found that this rate varies with the cycle of studies, showing an increase as the level of education advances, ranging from 2.9% in preschool education to 9.4% in the 3 ^rd^ cycle of basic education. No statistically significant differences were found in the distribution of anisometropia either between genders or between areas of residence. It was also found that myopic anisometropia was the most prevalent (46%), with a considerable increase in the 3 ^rd^ cycle of studies.

Table 5 summarises worldwide epidemiological data regarding studies in children and adolescents, which used the same criterion for anisometropia definition (IOD ≥ 1D).

**Table 5.** Anisometropia prevalence studies. TA - Total Anisometropia; SA - Spherical Equivalent Anisometropia; MA - Meridional Anisometropia.

Author/year	Location		Age (years)	Sample	Refractive test	Definition	Prevalence (%)
Ohlsson/2003 ^22^	America	Monterrey, Mexico	12-13	1035	Retinoscopy	AT ≥ 1.00	15
Dobson/2008 ^13^		Toronto, Canada	4-13	1041	Cycloplegic Autorefraction	SA ≥ 1.00 MA ≥ 1.00 TA ≥ 1.00	6.7 15 18.1
Deng/2012 ^5^		Boston, USA	5 12-15	395 312	Retinoscopy	AS ≥ 1.00	1.3 5.8
Quek/2004 ^23^	Asia	Singapore	15-19	946	N/Cycloplegic Autorefraction	SA ≥ 1.00	11.2
Yekta/2010 ^24^		Shiraz, Iran	7-15	1872	N/Cycloplegic Autorefraction	SA ≥ 1.00	2.6
Hu/2016 ^7^		Shandong, China	4-18	6025	Cycloplegic Autorefraction	SA ≥ 1.00 MA ≥ 1.00	7
Lee/2017 ^8^		Taiwan, China	8	23114	Cycloplegic Autorefraction	SA ≥ 1.00	3.7
Alrahili/2017 ^25^		Medina, Saudi Arabia	3-10	1893	N/Cycloplegic Autorefraction	TA ≥ 1.00	7.4
Hendriks/2009 ^4^	Europe	Maastricht, Netherlands	11-13	520	N/Cycloplegic Autorefraction	SA ≥ 1.00	4.6
Flitcroft/2020 ^11^		Ireland	6-7	362	Cycloplegic Autorefraction	SA ≥ 1.00	6.9
Huynh/2006 ^26^	Oceania	Sydney, Australia	6	1765	Cycloplegic Autorefraction	AS ≥ 1.00 AM ≥ 1.00	1.6 1.0

Comparing with others studies, some authors point to a frequency similar to the one found on this research, ^4^^,^^7^^,^^8^^,^^11^^,^^25^ others point to a lower frequency ^24^ and others still refer to a higher frequency. ^22^^,^^23^ Studies that included only children aged five and six years report rates of 1.3% and 1.6% (SA). ^5^^,^^26^ The frequency of SA, found in the present study, in preschool education (children from three to six years old) was 1%, this value being closer to those studies. Other studies included participants from 15 to 19 years old and report rates of 11,2% for SA. ^23^ For the 3 ^rd^ cycle of studies (average between 12 and 15 years of age), the present study indicated a frequency of 8.6% (SA) and according to the observed variation pattern, at a more advanced age and school stage, a higher rate is expected.

Geographical and methodological issues make it difficult to compare prevalence studies. There is a pattern of greater variability in studies on the Asian continent, where for an identical age group, there are records ranging from 2.5% ^24^ to more than 10%; ^7^ however this is the continent where more studies on the subject are found. In Europe, more similar results are found, 4,6% and 6,9%. ^4^^,^^11^ In Portugal, a study in children from 6 to 11 years old found a 0.7% rate (5 in 672) of uncorrected anisometropia, ^27^ similar to the 1.1% (8 in 749) found in the present study.

In the literature, the pattern of variation of anisometropia as a function of age and during the school period is not clear, presenting discordant results. Although many studies focus on children, it is common to have relatively wide age ranges. Most authors conclude that anisometropia varies with age, ^4^^,^^5^^,^^7^^,^^28^ although there are also studies where this relationship has not been found. ^10^^,^^13^ Studies on the subject, at school age, which showed an increasing prevalence with age were carried out in populations with a high frequency of myopia, a condition whose prevalence increases in adolescence. ^28^ On the other hand, longitudinal studies show that the prevalence of anisometropia increases after children start attending school. ^5^^,^^7^^,^^25^ Given these two lines that justify the variation of anisometropia during school age, it appears that they are related to each other, since the progress in the school path is accompanied by increasing age. In the present study, the anisometropia prevalence was also found to increase with advancement in the level of education. Consequently the age factor is also contributing to this situation, with myopic anisometropia being the one that most contributes to this variation pattern.

Regarding the influence of gender on anisometropia, contradictory data are found in the scientific literature. While in one study it is reported that the prevalence found was higher in males ^28^ in others it is reported that the prevalence rates are higher in females. ^10^^,^^23^ The results of the present study revealed a higher frequency in females (7.1%) than in males (5.3%), however these differences were not statistically significant. This finding is in line with the results of other authors. ^9^^,^^13^^,^^25^^,^^26^

The influence of living in rural or urban areas has also been the object of study by several researchers, considering the development of myopia and, consequently, myopic anisometropia, which is more pronounced in urban areas. ^7^^,^^8^^,^^10^ The present study did not prove whether the area of residence and the frequency of anisometropia were related. This parameter is highly dependent on the way in which each author classifies the area of residence, as rural or urban, and the limits of these regions are sometimes difficult to define. Also, living in a rural area and working in an urban area means that the daily experience of some populations turns out to be more urban, in both environments. Some studies show that lifestyle parameters, such as reading and writing habits and time spent indoors, contribute to the prevalence of anisometropia variation. ^7^^,^^8^^,^^10^^,^^11^

This study has several strengths. The study was carried out in children and adolescents in a school environment. This allows minimizing the potential bias that occurs in the sampling process and avoids overestimation of the problem, when the investigation is carried out in a clinical environment. For this study, spherical anisometropia and astigmatic anisometropia were considered. The latter being disregarded in several studies and there is a record that this anisometropia is the one that most varies in terms of ethnicity. ^9^

Certain limitations can be pointed out in this study. Firstly, the use of autorefraction without cycloplegia is highlighted, which is not the gold standard method of refraction. This fact limits the analysis of the distribution of the different types of refractive errors found in the population studied. Despite the use of an open field auto refractometer, recognized as an instrument with good agreement with cycloplegic retinoscopy and which eliminates the need for cycloplegia in children ^17^^,^^18^ tends to underestimate hyperopia. ^17^^,^^19^ However, for the present study, this situation does not weaken the conclusions, as the refraction was evaluated in an open field and binocularly, both eyes are exposed to the same conditions. In addition to that studies by other authors with the same methodology, point out a sensitivity of 100% for the diagnosis of anisometropia. ^19^ Secondly, the choice of the cut-off point for the classification of anisometropia is pointed out. The literature recommends considering an IOD of at least 1.00D, however the sensitivity studies of the auto refractometer used, recommend considering 1.25D. ^16^ Thirdly, the signalling of the area of residence of the participants as rural or urban is reported, since the limits of these regions were at times difficult to define. Noting also that being a study carried out in an area of the inner country, whose territorial classification is of low density, it is expectable that habits and behaviours are more uniform between rural and urban areas. This wouldn’t be the case if the same study had been carried out in an area of greater population density.

For future studies, data regarding reading habits and time spent outdoors will be collected, since these can be risk factors for the development of refractive errors.

## Conclusions

The present study estimated the prevalence of anisometropia in Portuguese children from preschool to the 3 ^rd^ cycle of basic education finding an occurrence rate of 6.1%. The results of this work also showed that the level of the study cycle and the spherical anisometropia are related, verifying that it is low in preschool education and higher in the 3 ^rd^ cycle of basic education. It was also found that, hyperopic anisometropia was more prevalent in younger children and that with the progress of the school path, myopic anisometropia predominated. Regarding anisometropia, 17% of the subjects were uncorrected, which can be associated to an absence of visual related symptoms due to a possible amblyopia. Taking into account the cases of uncorrected anisometropia, in our opinion the implementation of visual screening programs is essential for the timely detection and correction of possible eye problems. This course of action will lead to better development, learning and school outcomes.

## Data availability

### Underlying data

Dryad: Portuguese Children Refractive data - VER+ Project, https://doi.org/10.5061/dryad.h44j0zpm5. ^29^

This project contains the following underlying data:

• PortugueseChildrenRefractiveDataVERmaisProjectV2.xlsx

Data are available under the terms of the Creative Commons Zero “No rights reserved” data waiver (CC0 1.0 Public domain dedication).
