# Supplementary material for: Prevalence of anisometropia in children and adolescents
Source: F1000Res. 2022 Jun 8;10:1101. Originally published 2021 Nov 1. [Version 4] doi: 10.12688/f1000research.73657.4 (PMC8729023; doi:10.12688/f1000research.73657.4)
Supplement: Peer review history

# Peer review: Reviewer response for version 4

**DOI:** 10.5256/f1000research.134743.r140056

**Published:** 2023-07-12

**Authors:** Carla Lança

**Affiliations:** 1 Escola Superior de Tecnologia da Saúde de Lisboa (ESTeSL), Instituto Politecnico de Lisboa, Lisbon, Lisbon, Portugal; 2 Comprehensive Health Research Center (CHRC), Escola Nacional de Saúde Pública, Universidade Nova de Lisboa, Lisboa, Portugal

NA

Is the work clearly and accurately presented and does it cite the current literature?

Partly

If applicable, is the statistical analysis and its interpretation appropriate?

Partly

Are all the source data underlying the results available to ensure full reproducibility?

Yes

Is the study design appropriate and is the work technically sound?

Partly

Are the conclusions drawn adequately supported by the results?

Partly

Are sufficient details of methods and analysis provided to allow replication by others?

Partly

Reviewer Expertise:

Refractive errors and strabismus

I confirm that I have read this submission and believe that I have an appropriate level of expertise to confirm that it is of an acceptable scientific standard.

# Peer review: Reviewer response for version 3

**DOI:** 10.5256/f1000research.119923.r119238

**Published:** 2022-01-13

**Authors:** Carla Lança

**Affiliations:** 1 Escola Superior de Tecnologia da Saúde de Lisboa (ESTeSL), Instituto Politecnico de Lisboa, Lisbon, Lisbon, Portugal; 2 Comprehensive Health Research Center (CHRC), Escola Nacional de Saúde Pública, Universidade Nova de Lisboa, Lisboa, Portugal

The authors have revised the paper and addressed my previous comments. I still have 3 minor suggestions as described below. 

 Page 1 of 28: and page 7 of 28

- Please, delete “χ2(3) = 10.918;”

Page 5 of 28:

- Please, delete the final “e” from “componente”.
- Please, delete “and by applying the central limit theorem, it can be considered that the violation of the assumptions does not have serious consequences."

Is the work clearly and accurately presented and does it cite the current literature?

Partly

If applicable, is the statistical analysis and its interpretation appropriate?

Partly

Are all the source data underlying the results available to ensure full reproducibility?

Yes

Is the study design appropriate and is the work technically sound?

Partly

Are the conclusions drawn adequately supported by the results?

Partly

Are sufficient details of methods and analysis provided to allow replication by others?

Partly

Reviewer Expertise:

Refractive errors and strabismus

I confirm that I have read this submission and believe that I have an appropriate level of expertise to confirm that it is of an acceptable scientific standard.

# Response

**Published:** 2022-06-02

**Authors:** Amelia Nunes

**Affiliations:** Universidade da Beira Interior, Portugal

Dear reviewer,

 All corrections have been made.

 Thank you so much for your careful and valuable review.

# Peer review: Reviewer response for version 2

**DOI:** 10.5256/f1000research.79990.r102249

**Published:** 2021-12-24

**Authors:** Pedro M. Serra

**Affiliations:** 1 Research and Development Department, Ophthalmology Clinic Vista Sánchez Trancón, Badajoz, Spain

I would like to thank the authors for having implemented my recommendations in the current version of the manuscript. Generally, the manuscript improved considerably but it requires linguist editing.

**Specific comments**

***Abstract: ***

- I recommend using the word prevalent instead of “frequent”, it highlights the aim of the study.
- When possible avoid passive voice “ *An increase in anisometropia with the educational stage, was found in this study*”. May be better: This study found an increase in…

***Introduction:***

- “ *Anisometropia is an ocular disorder characterized by an interocular difference (IOD) in refractive error. It represents a specific refractive condition where the two eyes of an individual, with similar sociodemographic, environmental, and genetic influences, can have asymmetric eye growth*” Recommended - Anisometropia is an ocular disorder characterized by an interocular difference (IOD) in refractive error. It represents a specific refractive condition where the two eyes of an individual, can have asymmetric eye growth.
- “Scientific studies on the prevalence of anisometropia focus on children or adults, with less research being found in adolescence, table 1. Recommended: remove the word Scientific and place table 1 in the discussion.

***Methods:***

- I think it would be useful to indicate how rural and urban schools were segmented. For instance, say rural Schools were defined when settled in a living area with less than X thousand inhabitants.
- “Only two volunteers were excluded.” Be consistent with the terminology used, participants or volunteers?
- I believe Table 2 would be better placed in the results part.

**Discussion**

- The discussion needs to have the sentences shortened and the sentences need to be more objective.

Is the work clearly and accurately presented and does it cite the current literature?

Partly

If applicable, is the statistical analysis and its interpretation appropriate?

Yes

Are all the source data underlying the results available to ensure full reproducibility?

Yes

Is the study design appropriate and is the work technically sound?

Yes

Are the conclusions drawn adequately supported by the results?

Partly

Are sufficient details of methods and analysis provided to allow replication by others?

Partly

Reviewer Expertise:

Vision Sciences, Ophthalmic Optics.

I confirm that I have read this submission and believe that I have an appropriate level of expertise to confirm that it is of an acceptable scientific standard.

# Response

**Published:** 2021-12-30

**Authors:** Amelia Nunes

**Affiliations:** Universidade da Beira Interior, Portugal

Thank you so much for the review. We have made the changes accordingly based on the comments.

***Abstract:***

 I recommend using the word prevalent instead of “frequent”, it highlights the aim of the study.

**Response:** The wording was replaced in the abstract and in the remaining text.

 When possible avoid passive voice “ *An increase in anisometropia with the educational stage, was found in this study*”. May be better: This study found an increase in…

**Response:** The sentence was modified.

***Introduction:***

 “ *Anisometropia is an ocular disorder characterized by an interocular difference (IOD) in refractive error. It represents a specific refractive condition where the two eyes of an individual, with similar sociodemographic, environmental, and genetic influences, can have asymmetric eye growth*” Recommended - Anisometropia is an ocular disorder characterized by an interocular difference (IOD) in refractive error. It represents a specific refractive condition where the two eyes of an individual, can have asymmetric eye growth.

**Response:** The sentence was modified.

 “Scientific studies on the prevalence of anisometropia focus on children or adults, with less research being found in adolescence, table 1. Recommended: remove the word Scientific and place table 1 in the discussion.

**Response:** The word was removed and table 1 moved to the discussion section.

***Methods:***

 I think it would be useful to indicate how rural and urban schools were segmented. For instance, say rural Schools were defined when settled in a living area with less than X thousand inhabitants.

**Response:** That segmentation wasn’t done by this research team, it was provided by the municipalities representative. The following sentence was added to the sample characterization methods section “Rural and urban areas classification was based on the information provided by the municipalities, considering the residence area of each participant.” 

 “Only two volunteers were excluded.” Be consistent with the terminology used, participants or volunteers?

**Response:** The terminology was all changed to participants.

 I believe Table 2 would be better placed in the results part.

**Response:** In the first version of the manuscript, table 2 was in the results section. However since it presented data regarding the sample characterization, the other reviewer requested that it should be moved to the methods section.

**Discussion**

 The discussion needs to have the sentences shortened and the sentences need to be more objective.

**Response:** Several sentences have been changed in the discussion section.

# Peer review: Reviewer response for version 2

**DOI:** 10.5256/f1000research.79990.r102250

**Published:** 2021-12-10

**Authors:** Carla Lança

**Affiliations:** 1 Escola Superior de Tecnologia da Saúde de Lisboa (ESTeSL), Instituto Politecnico de Lisboa, Lisbon, Lisbon, Portugal; 2 Comprehensive Health Research Center (CHRC), Escola Nacional de Saúde Pública, Universidade Nova de Lisboa, Lisboa, Portugal

The paper is much improved, and the authors have addressed most of my comments. However, I still have a few minor comments:  

- Introduction: Table 1 should be moved to the discussion section. It is not common to see tables in the introduction section of scientific papers. The table needs a legend for the abbreviations mentioned. 
- Results: Table 5: The values of Z(adjust) do not add much to the table and the p-value is best. I suggest deleting the Z(adjust) values.
- Conclusions: The authors refer that screening programmes are necessary. However, only 8 of the 46 participants did not have a correction, meaning that the majority had been already diagnosed. The conclusion needs further refinement. 

Is the work clearly and accurately presented and does it cite the current literature?

Partly

If applicable, is the statistical analysis and its interpretation appropriate?

Partly

Are all the source data underlying the results available to ensure full reproducibility?

Yes

Is the study design appropriate and is the work technically sound?

Partly

Are the conclusions drawn adequately supported by the results?

Partly

Are sufficient details of methods and analysis provided to allow replication by others?

Partly

Reviewer Expertise:

Refractive errors and strabismus

I confirm that I have read this submission and believe that I have an appropriate level of expertise to confirm that it is of an acceptable scientific standard.

# Response

**Published:** 2021-12-30

**Authors:** Amelia Nunes

**Affiliations:** Universidade da Beira Interior, Portugal

Thank you so much for the review. We have made the changes accordingly based on the comments.

 Introduction: Table 1 should be moved to the discussion section. It is not common to see tables in the introduction section of scientific papers. The table needs a legend for the abbreviations mentioned.

**Response:**   Table 1 was moved to the discussion section (table and reference numbers will be updated)

 Results: Table 5: The values of Z(adjust) do not add much to the table and the p-value is best. I suggest deleting the Z(adjust) values.

**Response:**   Table 5, Z(adjust) deleted

 Conclusions: The authors refer that screening programs are necessary. However, only 8 of the 46 participants did not have a correction, meaning that the majority had been already diagnosed. The conclusion needs further refinement. 

**Response:** The last paragraph of the conclusion was changed to “Regarding anisometropia, 17% of the subjects were uncorrected, which can be associated to an absence of visual related symptoms due to a possible amblyopia. Taking into account the cases of uncorrected anisometropia, in our opinion the implementation of visual screening programs is essential for the timely detection and correction of possible eye problems. This course of action will lead to better development, learning and school outcomes.”

# Peer review: Reviewer response for version 1

**DOI:** 10.5256/f1000research.77322.r98419

**Published:** 2021-11-19

**Authors:** Pedro M. Serra

**Affiliations:** 1 Research and Development Department, Ophthalmology Clinic Vista Sánchez Trancón, Badajoz, Spain

**General comment**

 Thank you for considering my name as a reviewer of the manuscript titled “Frequency of anisometropia in children and adolescents”. I would like to congratulate the authors for their work in this field of vision sciences which has a special interest in countries where is a shortage of epidemiologic data in the field of vision.

 Regarding the manuscript, my feeling is that it requires additional work before it is considered for indexing. In this report I will cover the technical issues that in my opinion would improve the quality of the manuscript, however, I would recommend the author's linguistic editing of the final manuscript. Examples of linguistic improvement are very long sentences with multiple ideas and some lack of concise writing.

**Specific topics**

**Abstract:** Provide a statement defining the topic of research and a second sentence where you state the aim of the study (eg. This study aims to estimate the prevalence of anisometropia in the Portuguese population of children and adolescents.) I suggest changing the word frequency to prevalence throughout the text.

 Methods:

- Indicate the study design? (e.g. Observational Cross-sectional study)
- How did you build the sample?
- Describe the dependent and independent variables
- Remove any data from the methods

Results

- Use direct messages (e.g. No association was found between the presence of anisometropia with gender or area of residence)

Conclusion

- The first sentence should be deleted this is not a conclusion from the present study.

**Introduction:**

 I think the introduction requires some improvement

- 1 ^st^ paragraph – Provides a definition of the research topic
- “Anisometropia is an ocular disorder characterized by an interocular difference (IOD) in refractive error, representing a specific refractive condition insofar as the two eyes of an individual, with presumably similar sociodemographic, environmental and genetic influences, can have asymmetric eye growth.” Please reformulate this sentence, particularly remove the word presumably.
- 2 ^nd^ paragraph and 3 ^rd^ paragraph – mention the risk factors for anisometropia and the importance of assessing it
- 4 ^th^ paragraph- describes the instrument used. I would suggest moving this information to the methods
- 5 ^th^ paragraph- states the aim of the study.
- Missing information- the authors should mention the prevalence studies published, especially in Portugal, and compare with other European countries.  

**Methods:**

 Please include:

- Type of study
- Nature of the sample, how did you select the different groups? There are differences in the socioeconomic nature of the study sites? Please indicate how did you define Rural and Urban populations? (e.g. number of inhabitants covered by each school)?
- Please indicate specifically the region where this study was conducted, this is important for instance in future meta-analysis studies.
- Duration of the study (e.g September 2021 – November 2021)
- Could you add information about the use of spectacles, you could try to see if the anisometropia had already been identified.

Instrument description

- Include PlusOptix manufacturer city and country
- Provide the repeatability of the instrument (published)

This is just a comment - “ Students' data for which it was not possible to obtain refraction were excluded, due to technical issues associated with the performance of the instrument (presence of strabismus, opacities, retinal anomalies or when the refractive error exceeded the instrument's measurement limit - spherical measurement range or cylindrical from −7.00 to +5.00D) or due to lack of cooperation from the participant.”

 This is probably one of the most valuable pieces of information from the data collection and it is a shame not being presented.

**Data Analysis**

- Please indicate how the reader can interpret Thibos’ notation in regards to the J0 and J45.
- Say that the Cylinder component needs to be in negative form

Anisometropia Classification

- I suggest writing the formulas used for calculating the SA, MA, and sMA.

**Statistical Analysis**

**Results**

- Sample characterization – 1 ^st^ paragraph move it to the methods section.
- Refractive State
  - Please provide a table or figure (Box plot) with the refractive error information, where is possible to see the mean, standard deviation and range
  - Figure 1 has limited information, I suggest including the percentages in the table mentioned above
- Anisometropia
  - Please, use written numbers when placing them at the beginning of a sentence. (e.g Forty-six not 46)
  - Provide information about the level of anisometropia per type. Describe the mean, standard deviation, and range
  - Suggest creating a figure (bar plot with the percentages of anisometropia, total, young age group and older age group)

**Discussion**

- The authors found a statistically significant association between the frequency of myopic anisometropia and age/ scholarly. Could the authors extend the discussion on the etiology of myopic anisometropia, which probably implies an asymmetric eye growth? Discussing this point could help the authors to suggest actions/mechanisms to prevent anisometropia.
- Discuss the consequences of hyperopic anisometropia, use the values (mean and standard deviation) for arguing about the number of subjects in risk of developing amblyopia.

Is the work clearly and accurately presented and does it cite the current literature?

Partly

If applicable, is the statistical analysis and its interpretation appropriate?

Yes

Are all the source data underlying the results available to ensure full reproducibility?

Yes

Is the study design appropriate and is the work technically sound?

Yes

Are the conclusions drawn adequately supported by the results?

Partly

Are sufficient details of methods and analysis provided to allow replication by others?

Partly

Reviewer Expertise:

Vision Sciences, Ophthalmic Optics.

I confirm that I have read this submission and believe that I have an appropriate level of expertise to confirm that it is of an acceptable scientific standard, however I have significant reservations, as outlined above.

# Response

**Published:** 2021-11-30

**Authors:** Amelia Nunes

**Affiliations:** Universidade da Beira Interior, Portugal

We thank the reviewer for his time and very constructive comments. We appreciate the reviewers' voluntary contributions in the form of helpful comments that allowed us to strengthen our article.  We updated the article according to your comments.

**Specific topics**

**Abstract:** Provide a statement defining the topic of research and a second sentence where you state the aim of the study (eg. This study aims to estimate the prevalence of anisometropia in the Portuguese population of children and adolescents.) I suggest changing the word frequency to prevalence throughout the text.

**R:** The abstract was modified to include the topic and the aims “This research was developed to study the epidemiology of anisometropia. It aims to estimate the prevalence of anisometropia in Portuguese children and adolescents at various educational stages, studying its association with sociodemographic variables”

 Methods:

- Indicate the study design? (e.g. Observational Cross-sectional study)
- How did you build the sample?
- Describe the dependent and independent variables
- Remove any data from the methods

**R:** All information was included and data was removed. The sample was collected from several kindergartens and schools in the central region of Portugal. The central region was included in the abstract and more details were added to the methods section.

 Results

- Use direct messages (e.g. No association was found between the presence of anisometropia with gender or area of residence)

**R:** Section changed

 Conclusion

- The first sentence should be deleted this is not a conclusion from the present study.

**R:** Section changed

**Introduction:**

 I think the introduction requires some improvement

- 1 ^st^ paragraph – Provides a definition of the research topic
- “Anisometropia is an ocular disorder characterized by an interocular difference (IOD) in refractive error, representing a specific refractive condition insofar as the two eyes of an individual, with presumably similar sociodemographic, environmental and genetic influences, can have asymmetric eye growth.” Please reformulate this sentence, particularly remove the word presumably.
- 2 ^nd^ paragraph and 3 ^rd^ paragraph – mention the risk factors for anisometropia and the importance of assessing it
- 4 ^th^ paragraph- describes the instrument used. I would suggest moving this information to the methods
- 5 ^th^ paragraph- states the aim of the study.
- Missing information- the authors should mention the prevalence studies published, especially in Portugal, and compare with other European countries.  

**R: **All information was included. A new table (new table 1) was introduced containing prevalence studies. We did not find any studies regarding anisometropia prevalence in Portugal, in indexed research papers. We found an indexed research paper by Lança, Serra, and Prista in Portuguese children, which indirectly refers to the number of uncorrected anisometropic children, but the methodology is very different from ours, including the anisometropia classification criterium. However, the percentage of uncorrected anisometropia is similar. We considered this fact relevant and it was included in the discussion.

**Methods:**

 Please include:

- Type of study
- Nature of the sample, how did you select the different groups? There are differences in the socioeconomic nature of the study sites? Please indicate how did you define Rural and Urban populations? (e.g. number of inhabitants covered by each school)?
- Please indicate specifically the region where this study was conducted, this is important for instance in future meta-analysis studies.
- Duration of the study (e.g September 2021 – November 2021)
- Could you add information about the use of spectacles, you could try to see if the anisometropia had already been identified.

**R: **This information is important and was included to some extent in the methods section. Rural and Urban definitions were based on the information provided by the municipalities representative.

 Instrument description

- Include PlusOptix manufacturer city and country
- Provide the repeatability of the instrument (published)

This is just a comment - “ Students' data for which it was not possible to obtain refraction were excluded, due to technical issues associated with the performance of the instrument (presence of strabismus, opacities, retinal anomalies or when the refractive error exceeded the instrument's measurement limit - spherical measurement range or cylindrical from −7.00 to +5.00D) or due to lack of cooperation from the participant.”

 This is probably one of the most valuable pieces of information from the data collection and it is a shame not being presented.

**R:** Information included. Although a study reports repeatability of around 0,6D for MSE with the A09 model (Plusoptix Vision Screener: the accuracy and repeatability of refractive measurements using a new autorefractor) this and other studies are not focused on anisometropia, for which IOD MSE errors could be minimized. Another study with a different plus optix model, reports a sensitivity of 100% for anisometropia (A comparison of plusoptiX A12 measurements with cycloplegic refraction). We included this study in the introduction.

**Data Analysis**

- Please indicate how the reader can interpret Thibos’ notation in regards to the J0 and J45.
- Say that the Cylinder component needs to be in negative form

**R:** This information has been added

 Anisometropia Classification

- I suggest writing the formulas used for calculating the SA, MA, and sMA.

**R:** The formulas were added

**Statistical Analysis**

**Results**

- Sample characterization – 1 ^st^ paragraph move it to the methods section.

**R:** Paragraph moved

  

- Refractive State
  - Please provide a table or figure (Box plot) with the refractive error information, where is possible to see the mean, standard deviation, and range
  - Figure 1 has limited information, I suggest including the percentages in the table mentioned above

**R:** A new table (table 4) containing this data was added to this section

- Anisometropia
  - Please, use written numbers when placing them at the beginning of a sentence. (e.g Forty-six not 46)
  - Provide information about the level of anisometropia per type. Describe the mean, standard deviation, and range
  - Suggest creating a figure (bar plot with the percentages of anisometropia, total, young age group and older age group)

**R:** Sentence rewritten

 Mean, standard deviation, and range of anisometropia per type was added in table 4. An extra figure would duplicate the information.

 Information about the age group is already included in table 5, distributed by the study cycle which indirectly represents age groups. An extra figure would duplicate the information.

**Discussion**

- The authors found a statistically significant association between the frequency of myopic anisometropia and age/ scholarly. Could the authors extend the discussion on the etiology of myopic anisometropia, which probably implies an asymmetric eye growth? Discussing this point could help the authors to suggest actions/mechanisms to prevent anisometropia.

**R:** This is an excellent suggestion for future research. We can hypothesise that an excess of near-work activities, associated with incorrect posture, can lead to asymmetric eye growth. However, those factors were not controlled in the present study.

  

- Discuss the consequences of hyperopic anisometropia, use the values (mean and standard deviation) for arguing about the number of subjects at risk of developing amblyopia.

**R:** Only 2 uncorrected anisometropes were hyperopic, and since no BCVA data was collected, we feel that there are no sufficient data to support this kind of analysis. However, we agree that it is an excellent suggestion for future work.

# Peer review: Reviewer response for version 1

**DOI:** 10.5256/f1000research.77322.r98416

**Published:** 2021-11-12

**Authors:** Carla Lança

**Affiliations:** 1 Escola Superior de Tecnologia da Saúde de Lisboa (ESTeSL), Instituto Politecnico de Lisboa, Lisbon, Lisbon, Portugal; 2 Comprehensive Health Research Center (CHRC), Escola Nacional de Saúde Pública, Universidade Nova de Lisboa, Lisboa, Portugal

The study reports on the prevalence of anisometropia in a sample of Portuguese children and adolescents. The wording needs major revision. I have several comments as shown below.

**Title**

- I suggest replacing the word “frequency” by “prevalence”. This suggestion also applies throughout all sections of the manuscript.

**Abstract**

- Methods - Please replace “cycloplegic” by "cycloplegia”.
- Results - Please revise the wording of the following sentence “to state that the occurrence…”; The last sentence of the results it’s not clear and should be revised.

**Introduction**

- The first sentence is too long, and the wording needs to be revised. I suggest splitting it into two sentences. I have a similar comment for the third paragraph of the introduction section.
- There is no mention to previous studies on anisometropia prevalence. Also, there is the need to show more detailed results on the studies reporting risk factors and its association with anisometropia (e.g., sample size, age, p values, etc.).

**Methods**

- The methods section is incomplete. The methods section of a research paper provides the information by which a study's validity is judged. Therefore, it requires a clear and precise description of how an experiment was done, and the rationale for why specific experimental procedures were chosen. For example, more information is necessary on the sampling method and authors should describe their study design.

**Results**

- The first paragraph should be moved to the methods section.
- Table 2 – Replace “statistical test” by p value only (follow the same recommendation in the text results). Also, include a note referring to which comparisons are being made (for example, is it the distribution of children by cycles or the age by cycles?).
- At the time you are writing your article, you have already completed your study, so you should use past tense in your methodology and results section to record what you did and found (for example, “this difference was statistically significant”).
- Refractive state and anisometropia sections need to be re-written, as it is confusing to present two percentages at the same time. Perhaps the authors can consider showing separate sentences for those results.
- Influence of sociodemographic variables: the first sentence of this section should be moved to the statistical analysis; Delete “therefore this parameter is not in the table” and replace by the p value.  
- Table 3 – delete the X2 value and only present p value. Non-significant p values may be presented with only 2 decimal places.
- For risk factor analysis the multivariate adjusted analysis should be additionally shown.
- Figure 2. The colour of the legend is not clear, and it is difficult to distinguish the categories presented in the graph. The y axis wording needs revision.

**Discussion**

- Please, delete the 1 ^st^ sentence.
- The differences between prevalence of anisometropia in Asia and Europe need a more careful explanation. Environmental factors play a major role in the development of refractive errors, especially myopia.
- Another limitation of the study that was not referred is that factors such as reading habits or outdoor time were not collected and those may be risk factors for the development of refractive errors.
- Is this the first study in Portugal? Are your results comparable with other studies in Portugal?

**Conclusions**

- Please revise the wording on the sentence that includes the following: “we are of the opinion”.

Is the work clearly and accurately presented and does it cite the current literature?

Partly

If applicable, is the statistical analysis and its interpretation appropriate?

Partly

Are all the source data underlying the results available to ensure full reproducibility?

Yes

Is the study design appropriate and is the work technically sound?

Partly

Are the conclusions drawn adequately supported by the results?

Partly

Are sufficient details of methods and analysis provided to allow replication by others?

Partly

Reviewer Expertise:

Refractive errors and strabismus

I confirm that I have read this submission and believe that I have an appropriate level of expertise to confirm that it is of an acceptable scientific standard, however I have significant reservations, as outlined above.

# Response

**Published:** 2021-11-30

**Authors:** Amelia Nunes

**Affiliations:** Universidade da Beira Interior, Portugal

We would like to thank the reviewer for her valuable time and comments on our article; this will be very useful for us in future research and has allowed us to improve this document. We have made the required corrections.

**Title**

- I suggest replacing the word “frequency” by “prevalence”. This suggestion also applies throughout all sections of the manuscript.

**R:** The title was replaced

**Abstract**

- Methods - Please replace “cycloplegic” by "cycloplegia”.

**R:** The word was replaced

  

- Results - Please revise the wording of the following sentence “to state that the occurrence…”; The last sentence of the results it’s not clear and should be revised.

**R:** Sentence revised to “No statistical differences were found between genders or between areas of residence regarding the rate of anisometropia”;

 Last sentence revised to “Regarding spherical equivalent anisometropia, there is a pattern of variation that increases with the cycle of studies (�� ^2^_(3)_= 10.918; p = 0.012), with myopic anisometropia being the main contributor to this variation.”

**Introduction**

- The first sentence is too long, and the wording needs to be revised. I suggest splitting it into two sentences. I have a similar comment for the third paragraph of the introduction section.

**R: **The first sentence was split and wording revised to:” Anisometropia is an ocular disorder characterized by an interocular difference (IOD) in refractive error. It represents a specific refractive condition where the two eyes of an individual, with presumably similar sociodemographic, environmental and genetic influences, can have asymmetric eye growth.”

 The third paragraph split and wording revised to:” Although it is not clear what is the ideal age to perform the correction, in order to guarantee an ideal visual development and maturation, the early correction of anisometropia is important. ^11^ This prevents the development of other changes such as aniseikonia, amblyopia and strabismus ^3,12,13^ and even in small degrees (<1D) facilitates emmetropization. ^11^ It also improves quality of life, reducing or eliminating symptoms of visual discomfort.”

  

- There is no mention to previous studies on anisometropia prevalence. Also, there is the need to show more detailed results on the studies reporting risk factors and its association with anisometropia (e.g., sample size, age, p values, etc.).

**R:** These data were introduced in a new table, and the reference numbering was changed accordingly.

**Methods**

- The methods section is incomplete. The methods section of a research paper provides the information by which a study's validity is judged. Therefore, it requires a clear and precise description of how an experiment was done, and the rationale for why specific experimental procedures were chosen. For example, more information is necessary on the sampling method and authors should describe their study design.

**R:** The method section was completed including the following text “Data from the Portuguese Census 2011, shows a population of 319284 from 0 to 14 years old in the central region. For a confidence level of 95% and a 5% margin of error, taking into account that the prevalence of the studied condition is unknown, it was fixed at 50% to obtain a large enough sample size. The result was a minimum of 384 subjects. The data collection took place in 5 schools of the central region of Portugal, including all students that were authorized to participate by their legal guardians.”

**Results**

- The first paragraph should be moved to the methods section.

**R:** The paragraph was moved

  

- Table 2 – Replace “statistical test” by p value only (follow the same recommendation in the text results). Also, include a note referring to which comparisons are being made (for example, is it the distribution of children by cycles or the age by cycles?).

**R:** Done. This table was moved to the methods section, and the statistical analysis was deleted since it did not provide significant information.

- At the time you are writing your article, you have already completed your study, so you should use past tense in your methodology and results section to record what you did and found (for example, “this difference was statistically significant”).

**R:** Text rewritten to past tense.

  

- Refractive state and anisometropia sections need to be re-written, as it is confusing to present two percentages at the same time. Perhaps the authors can consider showing separate sentences for those results.

**R:** Both sections were rewritten to avoid double percentages. 

- Influence of sociodemographic variables: the first sentence of this section should be moved to the statistical analysis; Delete “therefore this parameter is not in the table” and replace by the p-value.  

**R:** The sentence was moved to the methods section. “Therefore..” was removed and the p-value information was added to the table.  

- Table 3 – delete the X2 value and only present p-value. Non-significant p values may be presented with only 2 decimal places.

**R:** X2 values deleted and p values with 2 decimal places

- For risk factor analysis the multivariate adjusted analysis should be additionally shown.

**R:** The multivariate analysis was added to the table.

  

- Figure 2. The colour of the legend is not clear, and it is difficult to distinguish the categories presented in the graph. The y axis wording needs revision.

**R:** Legend colour changed. Y-axis caption revised.

**Discussion**

- Please, delete the 1 ^st^ sentence.

**R:** Sentence deleted

- The differences between prevalence of anisometropia in Asia and Europe need a more careful explanation. Environmental factors play a major role in the development of refractive errors, especially myopia.

**R:** An extra sentence was added to explain to acknowledge the contribution of environmental factors “Some studies show that lifestyle parameters, such as reading and writing habits and time spent indoors, contribute to the prevalence of anisometropia variation. ^7,8,10,11^”

- Another limitation of the study that was not referred to is that factors such as reading habits or outdoor time were not collected and those may be risk factors for the development of refractive errors.

**R:** We recognise the relevance of these factors, and they will be included in future studies. Accordingly to your suggestions, the following sentence was added to the end of the text “For future studies, data regarding reading habits and time spent outdoors will be collected, since these can be risk factors for the development of refractive errors.”

- Is this the first study in Portugal? Are your results comparable with other studies in Portugal?

**R:** This is not the first study in Portugal, however in indexed research papers we couldn’t find any comparable studies for Portugal. We found an indexed research paper by Lança, Serra, and Prista in Portuguese children, which indirectly refers to the number of uncorrected anisometropic children, but the methodology is very different from ours, including the anisometropia classification criterium. However, the percentage of uncorrected anisometropia is similar. We considered this fact relevant and it was included in the discussion.

**Conclusions**

- Please revise the wording on the sentence that includes the following: “we are of the opinion”.

**R:** The sentence was revised and divided to: “Taking into account the high frequency of anisometropia found in 3 ^rd^ cycle students, in our opinion the implementation of visual screening programs at this age is essential for the timely detection and correction of possible eye problems.  This course of action will lead to better development, learning, and school outcomes at these ages.”
